# RGD-Dendrimer-Poly(L-lactic) Acid Nanopatterned Substrates for the Early Chondrogenesis of Human Mesenchymal Stromal Cells Derived from Osteoarthritic and Healthy Donors

**DOI:** 10.3390/ma13102247

**Published:** 2020-05-13

**Authors:** Cristina Rodríguez-Pereira, Anna Lagunas, Ignasi Casanellas, Yolanda Vida, Ezequiel Pérez-Inestrosa, José A. Andrades, José Becerra, Josep Samitier, Francisco J. Blanco, Joana Magalhães

**Affiliations:** 1 Unidad de Medicina Regenerativa, Grupo de Investigación en Reumatología (GIR), Instituto de Investigación Biomédica de A Coruña (INIBIC), Complejo Hospitalario Universitario de A Coruña (CHUAC), Sergas, 15006 A Coruña, Spain; cristina.rodriguez.pereira@sergas.es (C.R.-P.); fblagar@sergas.es (F.J.B.); 2 Centro de Investigaciones Científicas Avanzadas (CICA), Universidade da Coruña (UDC), As Carballeiras S/N, Campus de Elviña, 15071 A Coruña, Spain; 3 Networking Biomedical Research Center in Bioengineering, Biomaterials and Nanomedicine (CIBER-BBN), 28029 Madrid, Spain; alagunas@ibecbarcelona.eu (A.L.); icasanellas@ibecbarcelona.eu (I.C.); andrades@uma.es (J.A.A.); becerra@uma.es (J.B.); jsamitier@ibecbarcelona.eu (J.S.); 4 Institute for Bioengineering of Catalonia (IBEC), Barcelona Institute of Science and Technology (BIST), 08028 Barcelona, Spain; 5 Department of Electronics and Biomedical Engineering, University of Barcelona (UB), 08028 Barcelona, Spain; 6 Dpto. Química Orgánica, Universidad de Málaga-IBIMA, Campus de Teatinos s/n, 29071 Málaga, Spain; yolvida@uma.es (Y.V.); inestrosa@uma.es (E.P.-I.); 7 Centro Andaluz de Nanomedicina y Biotecnología (BIONAND), Parque Tecnológico de Andalucía, C/Severo Ochoa, 35, 29590 Campanillas, 29590 Málaga, Spain; 8 Cell Biology, Genetics and Physiology Department, Instituto de Investigación Biomédica de Málaga (IBIMA), University of Malaga (UMA), 29071 Málaga, Spain; 9 Departamento de Medicina, Facultad Ciencias de la Salud, Campus de Oza, Universidade da Coruña (UDC), Campus de Oza, 15006 A Coruña, Spain

**Keywords:** cell condensation, gap junctions, RGD-density, chondrogenic differentiation, osteoarthritis

## Abstract

Aiming to address a stable chondrogenesis derived from mesenchymal stromal cells (MSCs) to be applied in cartilage repair strategies at the onset of osteoarthritis (OA), we analyzed the effect of arginine–glycine–aspartate (RGD) density on cell condensation that occurs during the initial phase of chondrogenesis. For this, we seeded MSC-derived from OA and healthy (H) donors in RGD-dendrimer-poly(L-lactic) acid (PLLA) nanopatterned substrates (RGD concentrations of 4 × 10^−9^, 10^−8^, 2.5 × 10^−8^, and 10^−2^ w/w), during three days and compared to a cell pellet conventional three-dimensional culture system. Molecular gene expression (collagens type-I and II–COL1A1 and COL2A1, tenascin-TNC, sex determining region Y-box9-SOX9, and gap junction protein alpha 1–GJA1) was determined as well as the cell aggregates and pellet size, collagen type-II and connexin 43 proteins synthesis. This study showed that RGD-tailored first generation dendrimer (RGD-Cys-D1) PLLA nanopatterned substrates supported the formation of pre-chondrogenic condensates from OA- and H-derived human bone marrow-MSCs with enhanced chondrogenesis regarding the cell pellet conventional system (presence of collagen type-II and connexin 43, both at the gene and protein level). A RGD-density dependent trend was observed for aggregates size, in concordance with previous studies. Moreover, the nanopatterns’ had a higher effect on OA-derived MSC morphology, leading to the formation of bigger and more compact aggregates with improved expression of early chondrogenic markers.

## 1. Introduction

Osteoarthritis (OA) is a degenerative disease affecting the articular cartilage present at the movable joints, being characterized by cell stress and extracellular matrix (ECM) degradation, initiated by micro- and macro-injuries that activate maladaptive repair responses including pro-inflammatory pathways [1]. Given that nowadays there is still no cure, a need for clinical solutions is imperative. Regenerative medicine therapies for OA treatment, based on mesenchymal stromal cells (MSCs) and biomaterials have been of focus since the late 90s, and even though significant advances have been made, a successful guidance of the MSC chondrogenic differentiation process towards a stable adult chondrocyte phenotype has not yet been fully achieved [2,3].

Controlled nanoscaled topographies or surface nanopatterning have been proposed as potential tools for cell behavior guidance including adhesion, proliferation, and differentiation towards specific lineages via the regulation of membrane proteins [4,5]. Specific cell adhesion can be achieved via the formation of focal adhesions and triggered by the bioconjugation of ligands present in the ECM, such as fibronectin, and more specifically the arginine–glycine–aspartate (RGD) sequence, to the corresponding cell receptor, integrin [6]. Most research on the effect of RGD nanopatterns on MSC guidance has been focused on osteogenesis and adipogenesis, demonstrating the role of RGD nanospacing as an inherent regulator of stem cell differentiation [4,6,7,8]. On the other hand, fewer studies can be found on the effect of nanopattern tuning on MSC chondrogenesis, albeit these indicate that both nanopattern distribution and composition of group functionality can regulate the chondrogenic process [9,10].

Dendrimer nanopatterning is a bottom-up nanopatterning technique based on the self-assembly of absorbed dendrimers in low-charged surfaces in liquid-like order [11]. This technique allows the nanoscale control of surface functionalization on large areas, thus being fully compatible with cell culture procedures. In previous work, we have synthesized RGD-tailored first generation dendrimers (RGD-Cys-D1) that incorporate the fibronectin tripeptide sequence RGD, responsible for integrin-mediated cell adhesion [12]. Since dendrimers are 4–5 nm in diameter [12], although presenting up to eight copies of RGD, only one integrin (approximately 10 nm diameter) [13] will interact per dendrimer. Thereby, nanopattern configurations correspond to the available RGD distribution for cell adhesion. Nanopatterns of a range of biologically relevant local surface densities were thus obtained from different RGD-Cys-D1 initial concentrations in aqueous solution deposited on poly(L-lactic) acid (PLLA) substrates [12,14]. In this work, we have shown an association between local RGD surface density and adipose tissue (AT)-derived MSC chondrogenic commitment, demonstrating that an intermediate adhesiveness of cells resulted in larger cell condensates with enhanced early chondrogenic differentiation [14].

Condensation is a prerequisite for chondrogenic differentiation. In this cellular aggregation stage, cellular communication occurs determined by the expression of cell–cell adhesion molecules such as N-cadherin, neural cell adhesion molecule, or gap junctions [15]. Thus, these molecules, together with the ECM, must be strictly controlled to determine the size and shape of the condensations [16]. A high percentage of these cell-to-cell interactions are channels of the gap-junction (GJ) type, specifically those composed by connexin 43 (expressed by the gap junction protein alpha 1, GJA1) [17,18]. This protein has been described as being implicated in a great number of processes including chondrogenesis as well as in the pathogenesis of osteoarthritis [19]. In particular, our group was the first to demonstrate the expression of several GJ proteins in human cartilage as well as altered levels of different connexins including overexpression of GJA1 in OA-derived tissue [20].

Later, Schrobback et al. investigated the influence of gap junction-mediated intercellular contacts on bone marrow derived-MSCs chondrogenesis on alginate and microtissue models (restricted vs. induced cell–cell contacts), showing that a reduction in direct cell–cell contacts did not affect the chondrogenic process, however, blocking gap junctions compromised cell differentiation [21].

Hereby, this study was aimed to compare the chondrogenic differentiation potential of human BM-MSCs derived from OA or healthy (H) donors in uneven RGD-Cys-D1-PLLA nanopatterned substrates using a three-dimensional (3D) pellet culture system as a reference model to mimic prechondrogenic condensation, with a focus on the influence of gap-junction mediated cell-to-cell interactions in the chondrogenic differentiation process.

## 2. Materials and Methods

### 2.1. RGD-Cys-D1- Dendrimer PLLA Nanopatterned Substrate Production

A 2% m/v 95/5 L-lactide/DL-lactide copolymer solution (PLLA) (Corbion, Barcelona, Spain) in dry 1,4-dioxane (Panreac, Madrid, Spain) was deposited on 1.25 × 1.25 cm^2^ Corning glass microslides (Sigma-Aldrich, Madrid, Spain) and spin-coated at 3000 rpm for 30 s (Laurell Tech. Corp., North Wales, UK). Surface nanopatterning was conducted by immersing the spin-coated PLLA substrates in aqueous solutions of RGD-Cys-D1, for 16 h (pH = 5.6, T = 293 K), at the concentration of 4 × 10^−9^, 10^−8^, 2.5 × 10^−8^, and 10^−2^ w/w, followed by copious rinsing with water, as previously described [11]. Deionized water (18 MΩ·cm^−1^ Milli-Q, Millipore, Madrid) was used to prepare all solutions and for sample rinsing. All solutions were sonicated and filtered (Millex RB sterile filter, Merck Millipore, Madrid, Spain) prior to use. Ultraviolet light (UV) exposure for 15 min was used for sterilization before cellular experiments.

### 2.2. Bone Marrow-Mesenchymal Stromal Cells Isolation and Chondrogenic Differentiation

Human mesenchymal stromal cells (MSCs) were isolated from the bone marrow (BM) of femur heads obtained from seven patients (six females and one male, with a mean age of 88 ± 4.3), of which, four were osteoarthritic (OA) and three healthy (H), as classified by qualified clinical physicians, according to the Kellgren and Lawrence (K-L) radiological grading system [22]. The patients signed an informed consent agreement form prior to collection. The study was conducted in accordance with the Declaration of Helsinki and the protocol approved by the Galician Research Ethics Committee (Project Registry Code: 2014/019).

BM-MSCs were cultured in a basal medium (BM) composed by Dulbecco’s modified Eagle’s medium (DMEM, Lonza, Porriño, Spain) supplemented with 20% fetal bovine serum (Gibco, Madrid, Spain) and penicillin/streptomycin (10,000 IU/mL) (Gibco, Madrid, Spain) until 90% confluent. Pre-plating technique was performed to avoid remaining fibroblasts [23]. Cells used in all experiments were mycoplasma-free.

**Figure 1:**
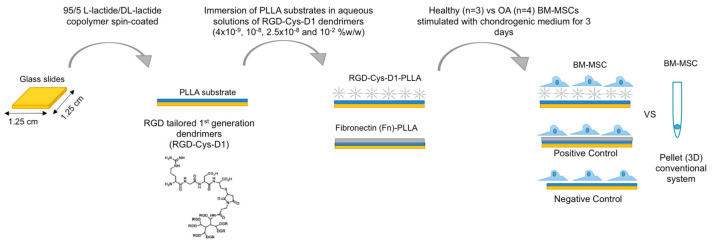
Schematic representation of the RGD-Cys-D1 PLLA nanopatterned substrates preparation process and cell seeding.

For chondrogenic differentiation studies (Figure 1), BM-MSCs derived from OA and H patients were seeded separately in a 12-well culture plate at densities of 5 x 10^3^ (for immunofluorescence) or 2.5 × 10^5^ (for gene expression analysis) cells per well, in RGD-Cys-D1 PLLA nanopatterned substrates, at different dendrimer concentrations (4 × 10^−9^, 10^−8^, 2.5 × 10^−8^, and 10^−2^ w/w). Fibronectin-coated PLLA (Fn-PLLA) (100 µg/mL) and untreated-PLLA were used as positive and negative controls, respectively.

Cells were incubated in a well-defined chondrogenic differentiation medium (CM) composed of DMEM supplemented with knockout serum (15%, Invitrogen, Barcelona, Spain), ascorbic acid (10 µL/mL), transferrin (6 µL/mL), dexamethasone (10 µM), retinoic acid (10^−7^ M) (all from Sigma-Aldrich, Madrid, Spain), and transforming growth factor beta 3 (10 ng/mL, Prospec-Tany TechnoGene, Deltaclon, Madrid, Spain), at 37 °C under 5% CO_2_ for three days. Cells seeded in Fn-PLLA under basal medium conditions were used as a negative control of chondrogenesis.

Finally, conventional 3D pellet culture was used as a reference model for chondrogenesis [24]. Briefly, 2.5 × 10^5^ cells, suspended in 500 μL CM, were centrifuged at 600 g, for 10 min, in 15 mL polypropylene conical tubes. Pellet-cells were incubated in the same conditions as our experimental model.

### 2.3. Immunofluorescence and Histology

PLLA-cell substrates were fixed in 4% (w/v) paraformaldehyde (Sigma-Aldrich, Madrid, Spain). BM-MSCs pellets were frozen in optimal cutting temperature (OCT) embedding matrix (Sakura, Barcelona, Spain). Immunofluorescence labeling was performed for collagen type-II (col-II) (1:50; Abcam, UK) and Cx43 (1:50; BD, Madrid, Spain). Fluorescein isothiocyanate (FITC) (1:10; DAKO, Barcelona, Spain) and ribulose-5-phosphate-3-epimerase (RPE) (1:20; DAKO, Barcelona, Spain) were respectively used as secondary antibodies, and 4’,6-diamidino-2-phenylindole (DAPI) (Sigma-Aldrich, Madrid, Spain) as counterstaining. Photographs were performed using the Olympus microscope (BX61, Olympus, Japan). Hematoxylin-Eosin (HE) and Safranin-O (SO) staining of the BM-MSCs pellets were performed in order to assess cell morphology and proteoglycan synthesis, respectively.

### 2.4. BM-MSC Aggregates and Pellet Areas

The area of BM-MSC aggregates formed in each experimental condition was calculated using ImageJ (Fiji version, U.S. National Institute of Health, Bethesda, MD, USA) [25] considering a cell aggregate as a group of at least five cells. The analysis was performed manually by selecting the cell aggregates (Selection tool) and determining their area with the Measure tool in fluorescence images of cells stained with DAPI. The results were presented as the mean of at least three cell aggregates. The area of each pellet formed using the conventional method was calculated following the same methodology, but using HE-stained micrographs. The results were presented as a mean of four OA- and three H-MSCs derived pellets, respectively.

### 2.5. Molecular Analysis

Total RNA (1 μg) was isolated from both PLLA-cell substrates and cell pellets, previously pulverized in a Mikro-Dismembrator (Retsch MM200, Retsch GmBH, Hann, Germany) using the Trizol method (Invitrogen, Madrid, Spain). RNA samples were treated with DNase and converted into cDNA using the SuperScript VILO cDNA synthesis kit (Invitrogen, Madrid, Spain).

Gene expression was measured by real-time quantitative reverse transcription-polymerase chain reaction (qRT-PCR) conducted in a LightCycler 480 Instrument (Roche, Madrid, Spain) using the LightCycler 480 Probes Master protocol. Amplification of mRNA was performed using custom-made primers for gap junction protein alpha 1 (GJA1), collagen type II (COL2A1), sex determining region Y-box9 (SOX9), tenascin (TNC), and collagen type I (COL1A1). RPL13a was used as the housekeeping gene (HKG) (Table 1).

The amplification program consisted of an initial denaturation at 95 °C for 10 min, followed by 45 cycles at 95 °C for 10 s, annealing at 60 °C for 30 s, and an extension at 72 °C for 1 s. Quantitative data were performed using qbase + software, version 3.1 (Biogazelle, Zwijnaarde, Belgium). Data were normalized against the expression values obtained for the positive control (Fn-PLLA) in healthy donor cells for each gene, which was assigned the value of 1, and measured as relative expression levels.

### 2.6. Statistical Analysis

All values were reported as mean ± standard deviation. Significant differences were judged using one-way analysis of variance (ANOVA) and the Student’s t-test for pairwise comparison, considering *p*-values < 0.05 statistically significant.

## 3. Results

### 3.1. BM-MSC Aggregates Formation and Areas

**Figure 2:**
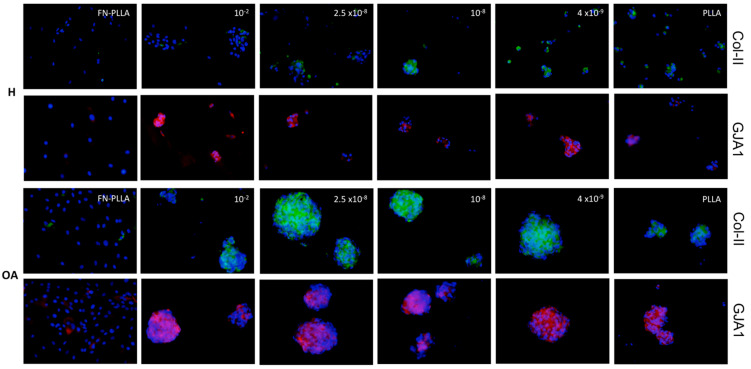
Collagen type-II (Col-II) and connexin 43 (expressed by the gap junction protein alpha 1, GJA1) immunofluorescence counterstained with DAPI, in healthy (H)- and osteoarthritic (OA)-derived bone marrow mesenchymal stromal cells (BM-MSCs) aggregates formed after three days, under chondrogenic medium (CM), in RGD-Cys-D1 PLLA nanopatterned substrates (10^−2^, 2.5 × 10^−8^, 10^−8^ and 4 × 10^−9^), fibronectin-coated PLLA (Fn-PLLA), and untreated PLLA (PLLA). Scale bar: 200 µm.

**Figure 3:**
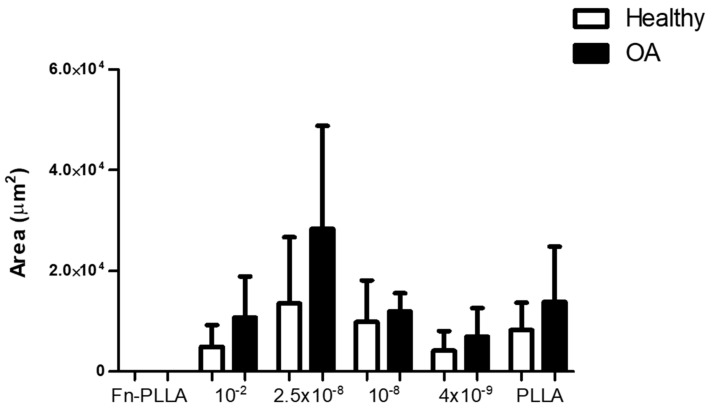
Area (units) of healthy- (H) and osteoarthritic (OA)-derived BM-MSC aggregates formed in RGD-Cys-D1 PLLA nanopatterned substrates (10^−2^, 2.5 × 10^−8^, 10^−8^, and 4 × 10^−9^), fibronectin-coated PLLA (Fn-PLLA), or untreated PLLA (PLLA) after three days, under chondrogenic medium (CM). Values are given as the mean of at least three aggregates with standard deviation.

RGD-Cys-D1-PLLA nanopatterned substrates supported the adhesion of OA- and H-derived BM-MSCs. Cell aggregation was observed in all functionalized surfaces, revealing different aggregation patterns and sizes (Figure 2) and showed that an increase in RGD-Cys-D1 dendrimer concentration, up to a concentration of 2.5 × 10^−8^, led to an increase in the cell aggregate area, decreasing for the highest concentration (10^−2^) (Figure 3). Even though this trend failed to reach statistical significance (*p* = 0.07), these results are concordant with previous findings.

Indeed, these results correlate with the increasing RGD density (surface adhesiveness) coated on PLLA substrates with dendrimers up to 2.5 × 10^−8^, which decreases at 10^−2^, because dendrimers at this concentration have been shown to aggregate in solution and adsorb on the carriers as clusters rather than individual particles [14]. Moreover, this distinct behavior is consistent with a RGD nanospacing threshold value around 70 nm, above which the cell adhesion process is delayed [26]. More specifically, H-derived MSC aggregate formation was observed in all conditions, except for Fn-PLLA (positive control), where cells were homogenously distributed in a monolayer.

Nonetheless, cell aggregates were either small (2.86 × 10^4^ to 0.39 × 10^4^ µm^2^) or appeared to be in the process of cell aggregation, but not fully compact (as can be distinctly observed for the 10^−2^ and 2.5 × 10^−8^ concentrations, top images, Figure 2). These values were in the range of the ones previously found for AT-MSCs [14]. On the other hand, OA-derived MSCs formed bigger (4.14 × 10^4^ to 0.22 × 10^4^ µm^2^) and compact aggregates.

**Figure 4:**
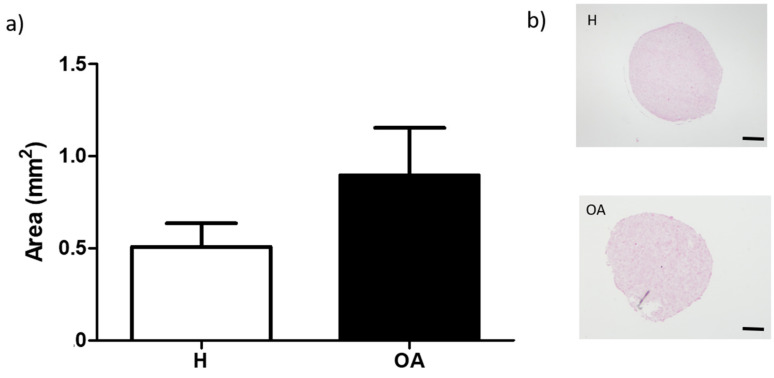
(**a**) Area (mm^2^) of H- and OA-derived BM-MSCs pellets in 3D-pellet conventional system after three days, under CM. Values are given as the mean of at least three donors with standard deviation. (**b**) Hematoxylin-Eosin (H-E) staining. Scale bar: 200 µm.

Untreated PLLA substrates (negative control) induced cell aggregation, more marked for OA-derived MSCs. In the negative control of chondrogenesis (basal medium) in Fn-PLLA substrates, cells were disposed in a confluent monolayer and did not form aggregates (Figure S1). Moreover, differences observed between the aggregate areas from OA- versus H-derived BM-MSCs seem to be independent of the substrate used, as this difference could also be appreciated in BM-MSCs pellets in the 3D conventional culture system (Figure 4).

### 3.2. Molecular Expression and Protein Synthesis

**Figure 5:**
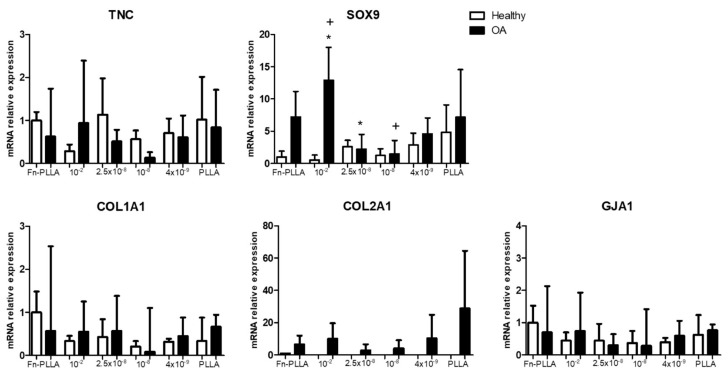
mRNA relative expression of TNC, SOX9, COL1A1, COL2A1, and GJA1 from H-(white) and OA-(black) derived BM-MSC aggregates formed in RGD-Cys-D1 PLLA nanopatterned substrates (10^−2^, 2.5 × 10^−8^, 10^−8^, and 4 × 10^−9^), fibronectin-coated PLLA (Fn-PLLA), and untreated PLLA (PLLA), after three days, under CM. Values are given as the mean of three donors with standard deviation. * + indicate *p* < 0.05.

Figure 5 represents the mRNA relative expression of cell aggregates from H- and OA-derived MSCs under the different conditions. Early chondrogenic markers, SOX9 and TNC, were upregulated for higher RGD-Cys-D1 dendrimer concentrations (2.5 × 10^−8^ in H and 10^−2^ in OA, although only significant for 10^−2^ vs. 10^−8^ and 10^−2^ vs. 2.5 × 10^−8^, in the latest) as well as in comparison with the controls (untreated PLLA and Fn-PLLA). COL1A1 expression followed a similar pattern such as other genes studied in H-derived cell aggregates, with an upregulation at 2.5 × 10^−8^, whilst in OA-derived cell aggregates, COL1A1 expression was practically unaltered and downregulated for all conditions when compared to Fn-PLLA.

The COL2A1 chondrogenic marker was only found expressed in OA-derived cell aggregates, with a similar expression trend as found for TNC and SOX9. Col-II protein expression was strongly synthesized when compared to the poorly stained H-derived cell aggregates (Figure 2). Even though the mRNA transcript level of the cartilage-related COL2A1 gene was non-detectable for the latest cells, the col-II protein was found accumulated in the extracellular matrix, which might be explained by the fact that other mechanisms regulate the protein’s abundance. As mRNA is prone to degradation, it is possible that mRNA has a rapid turnover while its protein has a higher half-life and remains accumulated [27].

GJA1 expression was upregulated for 2.5 × 10^−8^ regarding other dendrimer concentrations in H-derived cell aggregates, whilst showing a downregulation at this concentration in OA-derived cells. Cx43 was also observed at the protein level in both OA- and H-derived cell aggregates. In addition, OA-derived MSCs seeded in Fn-PLLA were poorly positive regarding H-derived cells that were negative (Figure 2). The same behavior was observed in the basal medium (Figure S1).

**Figure 6:**
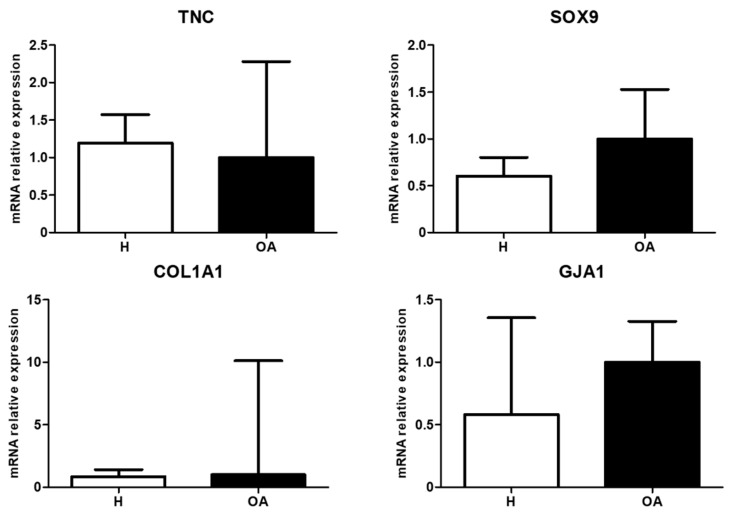
mRNA relative expression of TNC, SOX9, COL1A1, and GJA1 from H-(white) and OA-(black) derived BM-MSCs pellets, after three days, under CM. Values are given as the mean of three donors with standard deviation.

In the pellet system, comparison between H- and OA-derived MSCs indicated a downregulation of TNC and COL1A1 whilst an upregulation of GJA1 was observed. SOX9 showed a similar trend as observed for cell-aggregates formed in the different nanopatterns. In addition, no COL2A1 expression was detected (Figure 6).

**Figure 7:**
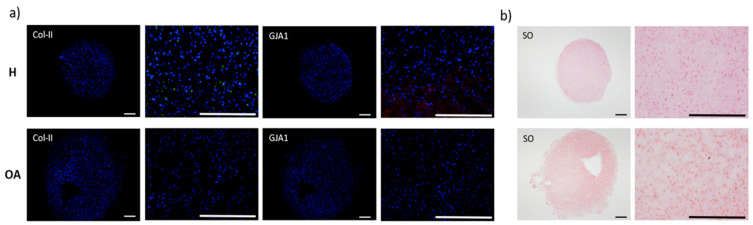
(**a**) Col-II and GJA1 immunofluorescence counterstained with DAPI. (**b**) SO staining for proteoglycans in H- and OA-derived BM-MSCs pellets, after three days, under CM. Scale bar: 200 µm.

Regarding protein expression in the pellets 3D-system, H-derived MSCs showed weak col-II staining that was limited to single cells and the absence of Cx43, whilst both markers were absent in pellets from OA-derived MSCs (Figure 7a). Nonetheless, SO staining, indicative of proteoglycans synthesis was found increased in the latest (Figure 7b).

## 4. Discussion

Cell condensation is a pivotal step during the early stages of chondrogenic differentiation. In this study, controlled nanopatterning of RGD-Cys-D1 over PLLA were used as bioactive substrates in order to evaluate RGD local surface density in the early chondrogenic differentiation of MSCs isolated from OA patients. Previous work demonstrated that tunable local RGD-Cys-D1 densities could be obtained as a function of the initial RGD-Cys-D1 dendrimer concentration, creating uneven distribution patterns of these first generation dendrimers, on PLLA coated glass surfaces [12]. Thereafter, together with our co-workers, we have shown that RGD-Cys-D1 PLLA nanopatterned substrates supported AT-MSC early chondrogenesis by stimulating the aggregation of the cells, followed by the maturation of focal adhesions that formed between them [14].

Whilst AT-MSCs are widely used and accepted, cellular models that can better resemble pathologic conditions are preferred, so we hereby intended to replicate the study conditions using human BM-MSC derived from OA and H donors. In addition, previous results from our group focused on cell-to-cell contact and cell communication in cartilage explant models showed higher expression of GJA1 in OA [20], this being associated with chondrocyte dedifferentiation [28]. Therefore, we intended to verify if there could be a dependency on connexin 43 and collagen-type II during a pre-chondrogenic phase of the differentiation process.

We found that already within three days after chondrogenic induction, MSCs seeded in RGD-Cys-D1 PLLA nanopatterned substrates condensed into cell aggregates and started a chondrogenic differentiation process (more pronounced for OA-derived MSCs). This was shown to be related to the presence and density of RGD-Cys-D1, suggesting 2.5 × 10^−8^ as an optimal dendrimer concentration, with higher expression of the early chondrogenic marker SOX9, which is consistent with previous observations using other MSC sources [14]. Moreover, our results support a higher specificity of RGD peptide compared with Fn-PLLA, having the latest induced a monolayer-type behavior and no observed aggregation (Figure 2 and Figure 3).

In addition, the cell–protein–material interface has been reported to affect cell migratory behavior, and differences in fibronectin conformation impact cell responses [29,30,31]. Even though we observed aggregation in untreated PLLA, this might be related to the polymer’s hydrophobicity, which causes cells to cluster in the presence of a chondrogenic medium, as reported for other positively charged polymers [32,33]. Other in-house studies using PLLA coated surfaces under basal medium showed no cell aggregation (data not shown).

Moreover, RGD-Cys-D1 PLLA nanopatterned substrates favored chondrogenesis regarding the conventional pellet culture system, supported by the synthesis of collagen type-II and an increased cell-to-cell contact network given by the presence of connexin-43, both on the gene (Figure 5 and Figure 7) and protein levels (Figure 2 and Figure 6), which could support the role of RGD-mediated adhesion via integrins that trigger cell arrangement and size, favoring a pre-chondrogenic state on cell aggregates [34,35,36,37,38]. This also supports previous findings [12,14].

Another interesting outcome of this study were the differences found between H- and OA-derived MSCs. On one hand, the increased expression of both GJA1 and COL2A1 in OA-derived MSC was in disagreement with other studies that showed a link between overexpression of GJA1 in OA chondrocytes and a compromised chondrogenic capability [20]. On the other hand, the downregulation of COL1A1 and GJA1 and absence of COL2A1 in H-derived aggregates may indicate an early migration phase that is RGD-dependent in which cells resemble a more undifferentiated state [34,39,40].

Furthermore, differences found could be related to the expression of different patterns of membrane proteins present in either OA- or H-derived cells, although these have not been considered in this study [41,42,43]. Finally, both OA and aging effects have been shown to impact the chondrogenic potential of MSCs. Even though OA-derived cells have reportedly been demonstrated to undergo chondrogenesis [44], these have rarely shown outperformance, as opposed to our findings. Nonetheless, there is still controversy and a need for studies using clearly defined OA progression criteria and age-matched control subject groups that can further deepen our knowledge for their clinical relevance.

## 5. Conclusions

The results obtained showed that RGD-Cys-D1 PLLA nanopatterned substrates supported the formation of pre-chondrogenic condensates from OA- and H-derived MSCs. OA-derived MSCs cultured in nanopatterned substrates formed bigger and more compact aggregates with improved collagen-II expression (both at gene and protein level) regarding either H-derived MSCs cultured in nanopatterned substrates or cell pellet conventional culture system, which support the use of the hereby proposed RGD-Cys-D1 PLLA nanopatterned substrates over the conventional pellet system in disease models.

## Figures and Tables

**Table 1:** Custom made primers.

Genes	Forward	Reverse	Probe	GeneBank Accession n°
CX43	gcctgaacttgccttttcat	ctccagtcacccatgttgc	88	NM_000165.4
COL2A1	tggtgctaatggcgagaag	cccagtctctccacgttcac	4	NM_001844.4
SOX9	gtacccgcacttgcacaac	tcgctctcgttcagaagtctc	61	NM_000346.3
COL1A1	ctggccccattggtaatgt	accagggaaaccagtagcac	1	NM_000088.3
TNC	ggtacagtgggacagcaggt	cccctttgtaggacagagca	9	NM_002160.3
RPL13A	caagcggatgaacaccaac	tgtggggcagcatacctc	28	NM_012423.3

## Supplementary Materials

The following are available online at https://www.mdpi.com/1996-1944/13/10/2247/s1, Figure S1: Col-II and GJA1 immunofluorescence counterstained with DAPI, in H- and OA-derived BM-MSCs for three days under basal medium in fibronectin-coated PLLA (Fn-PLLA). Scale bar: 200 µm.
