# Supplementary material for: RGD-Dendrimer-Poly(L-lactic) Acid Nanopatterned Substrates for the Early Chondrogenesis of Human Mesenchymal Stromal Cells Derived from Osteoarthritic and Healthy Donors
Source: Materials (Basel). 2020 May 13;13(10):2247. doi: 10.3390/ma13102247 (PMC7287591; doi:10.3390/ma13102247)
Supplement: Supplementary file 1 [file materials-13-02247-s001.pdf]

materials

MDPI

Supplementary Materials

RGD-Dendrimer-Poly(L-lactic) Acid Nanopatterned Substrates for the Early Chondrogenesis of Human Mesenchymal Stromal Cells Derived from Osteoarthritic and Healthy Donors

Cristina Rodríguez-Pereira [1,2], Anna Lagunas [3,4], Ignasi Casanellas [3,4,5], Yolanda Vida [6,7], Ezequiel Pérez-Inestrosa [6,7], José A. Andrades [3,8], José Becerra [3,7,8], Josep Samitier [3,4,5], Francisco J. Blanco [1,2,9] and Joana Magalhães [1,2,3,*]

1 Unidad de Medicina Regenerativa. Grupo de Investigación en Reumatología (GIR). Instituto de Investigación Biomédica de A Coruña (INIBIC). Complejo Hospitalario Universitario de A Coruña (CHUAC). Sergas, 15006 A Coruña, Spain; cristina.rodriguez.pereira@sergas.es (C.R.-P.); fblagar@sergas.es (F.J.B.)
2 Centro de Investigaciones Científicas Avanzadas (CICA), Universidade da Coruña (UDC). As Carballeiras S/N, Campus de Elviña, 15071 A Coruña, Spain
3 Networking Biomedical Research Center in Bioengineering, Biomaterials and Nanomedicine (CIBER-BBN), 28029 Madrid, Spain; alagunas@ibecbarcelona.eu (A.L.); icasanellas@ibecbarcelona.eu (I.C.); andrades@uma.es (J.A.A.); becerra@uma.es (J.B.); jsamitier@ibecbarcelona.eu (J.S.)
4 Institute for Bioengineering of Catalonia (IBEC), Barcelona Institute of Science and Technology (BIST), 08028 Barcelona, Spain
5 Department of Electronics and Biomedical Engineering, University of Barcelona (UB), 08028 Barcelona, Spain
6 Dpto. Química Orgánica, Universidad de Málaga-IBIMA, Campus de Teatinos s/n, 29071 Málaga, Spain; yolvida@uma.es (Y.V.); inestrosa@uma.es (E.P.-I.)
7 Centro Andaluz de Nanomedicina y Biotecnología (BIONAND). Parque Tecnológico de Andalucía. C/Severo Ochoa, 35, 29590 Campanillas, 29590 Málaga, Spain
8 Cell Biology, Genetics and Physiology Department, Instituto de Investigación Biomédica de Málaga (IBIMA), University of Malaga (UMA), 29071 Málaga, Spain;
9 Departamento de Medicina, Facultad Ciencias de la Salud, Campus de Oza, Universidade da Coruña (UDC). Campus de Oza, 15006 A Coruña, Spain
* Correspondence: joana.cristina.silva.magalhaes@sergas.es; Tel.: +34-981-176-413

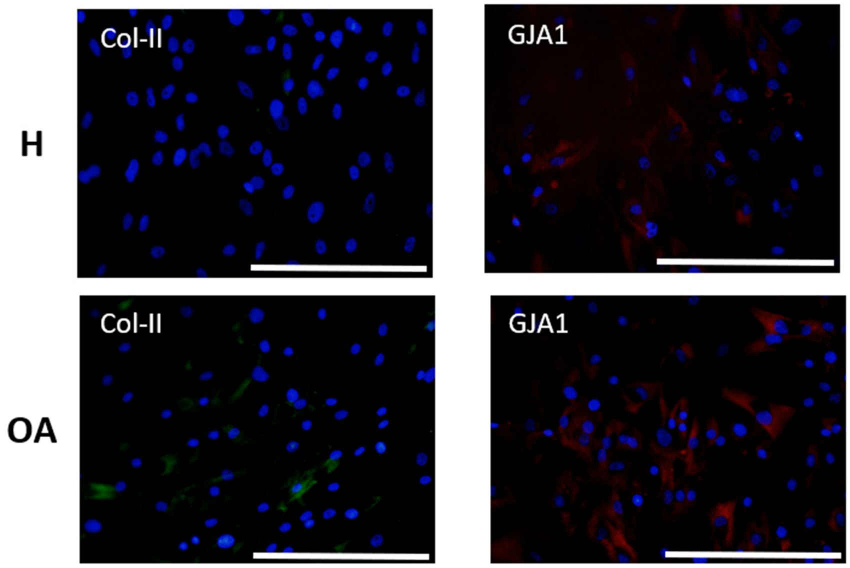

Figure S1. Col-II and GJA1 immunofluorescence counterstained with DAPI, in H- and OA-derived BM-MSCs, during 3 days, under basal medium, in fibronectin-coated PLLA (Fn-PLLA). Scale bar: 200 μm.

© 2020 by the authors. Submitted for possible open access publication under the terms and conditions of the Creative Commons Attribution (CC BY) license (http://creativecommons.org/licenses/by/4.0/).
